# Research on the Impact of Actual Tax Bearing Rate on the Financial Performance of Enterprises

**DOI:** 10.3389/fpubh.2022.940173

**Published:** 2022-08-08

**Authors:** Yao Dong, Chen Liang, Zhong Wanyin

**Affiliations:** School of Finance and Accounting, Chengdu Jincheng College, Chengdu, China

**Keywords:** actual tax bearing rate, pharmaceutical industry, financial performance, financial, non-state-owned

## Abstract

This paper is based on financial data analysis of 241 listed pharmaceutical manufacturing enterprises in China, we study the different effects of different tax reduction policies on the financial performance of enterprises in the short term and long term. Through the verification of practical examples, it could be found that the implementation of a tax reduction policy can indeed play a positive role in the financial performance of enterprises, and the reduction of income tax significantly improves the short-term financial performance of enterprises. However, the tax reduction of turnover tax significantly enhances the long-term financial performance of enterprises, and the effect of tax reduction on the financial performance of state-owned enterprises is better than that of non-state-owned enterprises. Generally speaking, tax reduction policy has a better effect on the financial performance of non-state-owned enterprises and enterprises in central and western regions than state-owned enterprises and enterprises in eastern regions.

## Introduction

### Research Background and Significance

At the end of 2019, COVID-19 began to spread in China. With the rapid response of the central government and appropriate measures, the epidemic has been contained. However, due to the impact of COVID-19, most of the enterprises has delayed the resumption of work, which lead to severe damage to the profits of many enterprises, even bankruptcy. Therefore, in order to reduce the losses suffered by enterprises as much as possible and help enterprises to get through the difficulties, the state has introduced a series of tax reductions and fee reduction policies. On March 3, 2020, Premier Li Keqiang presided over the executive meeting of the State Council and deployed the “six stable” work coordination mechanism, which decided to increase the intensity of tax reduction and fee reduction. On the same day, at the press conference of the joint defense mechanism of the State Council, director general Fu Jinling of the social security department of the Ministry of Finance introduced that this year's social insurance premium would reduce tax bearing for enterprises by more than 1 trillion yuan.

In this instant tax reduction policy, the government distinguishes the types and scales of different enterprises and take short-term emergency policies from the two various aspects of value-added tax and enterprise income tax. In fact, since the world financial crisis in 2008, in order to alleviate the impact of the financial crisis, maintain the regular operation and production of enterprises, stabilize the employment rate and maintain the growth rate of economic development, the Chinese government has been implementing tax reduction as an essential and effective policy. From “structural tax reduction” to “inclusive tax reduction,” more and more enterprises enjoy the dividend brought by the policy. As shown in Table 1, the main tax reduction policies since 2008 have been sorted out, from which it can be seen that the corresponding policies issued by the Chinese government on tax reduction are more and more frequent, powerful, and extensive.

**Table 1:** Major tax reduction in recent years.

**Year**	**Region**	**Main measures**
2008		First proposed “structural tax reduction”, 15 percent income tax rate shall be applied to enterprises that have obtained the “high-tech enterprise certificate”.
2009	Some regions	Start to implement structural tax reduction and “value-added tax transformation”.
2010	Some regions	Continue the implementation of the transformation reform of value-added tax, and the promotion of the reform of resource tax, etc.
2011	Some regions	Continue the implementation of structural tax reduction policies, the increment in personal income tax deductions from salaries and wages, continue the reduction import tariffs on certain commodities, increment in the threshold for value-added tax and business tax, and the implementation of preferential income tax policies for some small and small-profit enterprises, carried out trials in Shanghai to replace business tax with value-added tax in the transportation industry and some modern service industries.
2012	Some regions	Steady progress is made in piloting the change of business tax to value-added tax, continue to increase the value-added tax and threshold for business tax, and implement preferential income tax policies for small and micro enterprises.
2013	Some regions	Acceleration in the trial of “business tax to value-added tax” trials (develop the “business tax to value-added tax” pilot in the transportation industry and some modern service industries), expand the scope of resource tax ad valorem calculation, Expand the scope of additional deductions for research and development expenses, etc.
2014	Whole country	Preferential income tax for small and micro enterprises, simplified VAT levy rate, improve fixed assets to accelerate depreciation.
2015	Whole country	Further expansion in the scope of small and low-profit enterprises that levy 50% of corporate income tax.
2016	Whole country	Fully promote the “change of business tax to value-added tax”, the phased reduction of the social security provident fund rate, and the expansion of the scope of exemption of 18 administrative fees.
2017	Whole country	Continue to promote the “change of business tax to value-added tax”, expand the scope of small and micro enterprises that enjoy preferential corporate income tax, increase the pre-tax deduction ratio for R&D expenses of technology-based SMEs, etc.
2018	Whole country	Reduce VAT rates, unify small-scale taxpayer standards, expand deductions, increase deductions for employee education expenditures, accelerate depreciation of fixed assets, additional deductions for research and development expenses, relax conditions for technology-based enterprises in the start-up period, etc.
2019	Whole country	Personal income tax reform, inclusive tax reduction and exemption policies for small and micro enterprises, deepen VAT reform, reduce social insurance rates, support preferential tax policies for entrepreneurship and innovation, etc.

In the past ten years, the tax reduction policy has been implemented more and more comprehensively and deeply in China. This policy is not only an important measure of “supply-side reform,” but also a massive push for enterprises to continuously “reduce costs” and “make up for shortcomings.” So, what is the effect of the implementation, and what is the role of different tax reduction policies?

According to the previous literature, the main limitations of the research are as follows: First, at present, most articles mainly study equipment manufacturing industry, logistics industry, construction industry and other industries, and there are few literatures analyzing from the perspective of medical manufacturing industry; Second, in most of the literature, the tax categories selected for the study of tax reduction policies are relatively single, and mainly involve in the value-added tax reform, while the impact of different taxes is still relatively small; Thirdly, in many pieces of literature, the evaluation of the financial performance of enterprises start from short-term financial performance, but seldom combined short-term and long-term financial performance for analysis.

The innovation of this research lies in: First, A-share listed pharmaceutical manufacturing enterprises are selected as the research object; second, the impact of actual tax bearing rate of income tax and actual tax bearing rate of a turnover tax on financial performance is comprehensively compared; third, the analysis of financial performance is carried out by selecting different indicators to measure by distinguishing short-term and production period.

The significance of this study lies in: First, through the research of pharmaceutical manufacturing enterprises, we can know the actual effect of tax reduction policy on the pharmaceutical industry operation and enterprise operation and production, which will help policy-makers better grasp the direction of their policies; second, through the analysis of the specific impact of different tax reduction policies, we can know the different effects brought about by different policy tools, which will be beneficial to policymakers; third, through the research on the short-term and long-term performance of financial performance, we can make financial arrangements for decision-makers to make better use of policy benefits.

### Literature Review

At present, a large number of studies have focused on the impact of tax reduction on the financial performance of enterprises, but the conclusions of these studies are not uniform. In the view of some scholars, tax reduction has a significant role in promoting financial performance, while some scholars believe that there are differences in the effect of different tax reductions:

Caixia (1) select a sample of A-share listed companies in Shanghai and Shenzhen stock market and show empirically that the higher the cost of corporate income tax, the weaker the profitability (ROE) of the company; Chang et al. (2) take A-share listed companies of Shanghai Stock Exchange and A-share listed companies of Shenzhen Stock Exchange as samples, they use the actual tax bearing rate to measure the tax burden, and conclude that the tax burden has a significant negative correlation with the financial performance (ROE); Meiling (3) chooses A-share listed companies in Shanghai and Shenzhen stock markets for research, the results show that the tax burden is significantly negatively correlated with financial performance (ROA); Jun (4) combines case study and empirical study to research the impact of fiscal and tax subsidies on BYD's financial performance (ROA), and finds that income tax incentives can effectively improve financial performance; Xi (5) conducts an empirical analysis on the real estate development industry, and the research finds that “business tax to value-added tax” opening up the upstream and downstream deduction chain of the industry, which is conducive to the reduction of tax bearing of enterprises, thus improving the financial performance (ROE and ROA) of enterprises; Xiulian (6) establishes a regression model by using panel data of the real estate industry, according to his research results, it can be found that “business tax to value-added tax” has an impact on financial. Ran (7) studies the listed companies of the high-tech service industry, and the results show that the “business tax to value-added tax” improving the financial performance (ROE) by using the PSM-DID method; Qiping (8) studies the situation of high-tech enterprises after “business tax changed to value-added tax” by using the DID method, the results show that the advantages formed by the detailed division of labor promote financial performance (ROE) improved significantly; Chunke (9) researches a total of 50 enterprises listed on the Shanghai Stock Exchange, and the results show that “business tax to value-added tax” has a significant impact on the financial performance of enterprises performance (operating profit, asset-liability ratio and asset turnover ratio) have an increasing role; Yumei et al. (10) study the panel data of listed real estate companies and find that after “business tax changed to value-added tax,” financial performance (ROE) has indeed improved; Nafei and Lin (11) conduct a study based on the data of 31 listed agricultural companies by using multiple regression model, the research show that tax preference has an effect on the financial performance of agricultural listed companies performance (ROA) has a significant positive impact.

In addition, some scholars find that after the implementation of the tax reduction policy, the impact on the financial performance of enterprises is not obvious, or even has a negative impact. In some industries, the financial performance is gradually picking up after a certain number of years:

Yupin and Rui (12) conduct a study on logistics enterprises in Shanghai and Shenzhen stock markets, after the “business tax changed to value-added tax,” the overall tax bearing decreases, in logistics enterprises, the financial performance (ROE) decreases due to the general over investment in fixed assets; Yongqing (13) finds that “business tax changed to value-added tax” has an impact on the financial performance of modern service industry, performance (ROE) shows adverse effects in the same year, but improves year by year with the promotion of reform; Xiaoshuang (14) takes tourism companies as the research object, and finds that “business tax to value-added tax” would have a negative effect on the financial performance (ROE) of tourism service industry through case analysis; Yudi (15) reveals that after the implementation of “business tax to value-added tax”, the real estate enterprise financial performance (ROE) has declined, which may be due to the fact that real estate enterprises generally do not tend to update fixed assets, resulting in low efficiency of policy utilization; Guojuan (16) conducts an empirical analysis on Listed Companies in the construction industry, and obtains the financial effect of “business tax replacing value-added tax” through multiple linear regression analysis performance (ROA, ROE) has a negative correlation, but in a short period of time, the policy effect has not been clearly reflected, and the impact on non-state-owned listed companies in the construction industry is more significant than the state-owned ones; Dan (17) selects Ningbo Port Co. Ltd. as the sample, due to the reduction of deductible items and the unclear scope of Taxation, etc., it leads to the change of “business tax to value-added tax,” financial performance (ROA, ROE) has declined; Qin and Jing (18) accords to panel data of 74 information technology service companies in Shanghai and Shenzhen stock markets, using the DID method, finds that the financial performance (ROE) of enterprises in the year of “business tax to value-added tax” decline, but gradually pick up over time.

Based on the above literature conclusions, we can see that tax bearing does have a significant impact on the financial performance of enterprises. In the view of most scholars, they all think that tax cuts will bring improvement of financial performance, but a few scholars think that tax cuts cannot play a rapid positive effect in the short term, which need to be verified for a period of time. In the research of tax reduction, most of them is about “business tax to value-added tax,” while the research on income tax is relatively less. In terms of the selection of financial performance indicators, most of the researches choose short-term financial performance as their research indicators, while the researches on long-term financial performance indicators of enterprises are particularly rare. However, for the general policy effect, it is difficult to play an obvious effect in a short time. From the national level, the implementation of the policy needs constant exploration and adjustment, while for enterprises, they need to understand and adapt to the policy constantly. Based on this situation, this paper makes a comprehensive study of different tax reduction policies, short-term and long-term financial indicators, and effect lagging items in order to supplement and improve the above deficiencies.

## Theoretical Analysis and Hypothesis

From the micro point of view, the tax reduction is actually equivalent to the indirect financial subsidies given by the state to enterprises, which can be used by enterprises to expand production scale, introduce talents, technological innovation and other aspects (19, 20). Through tax reduction, the second optimal allocation of resources can be realized. At the same time, tax activities will generate tax costs and non-tax costs, which will directly affect the cash flow of enterprises, and then affect the decision-making of enterprise leaders. In addition, at the macro level, the implementation of a tax reduction policy can reflect the continuous reform of the state's tax system, which has a huge role in promoting the optimization and adjustment of industrial structure and the transformation and upgrading of economic development momentum (21–23).

**Figure 1:**
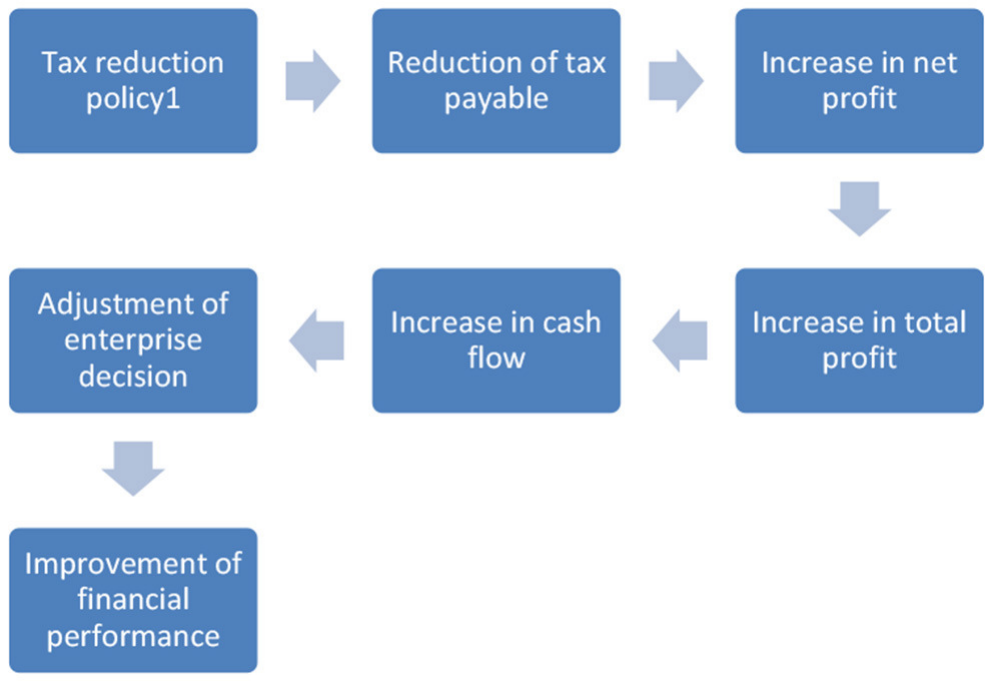
Mechanism of tax reduction.

As shown in Figure 1, from the perspective of its mechanism, the implementation of tax reduction policy is mainly focused on the government's targeted tax relief for key industries and fields to a certain extent, which reduces the tax burden of enterprises, increases the net profit and total profit, and expands the cash flow of enterprises. Therefore, the enterprise decision-makers can use it in all aspects of production and operation, so as to improve the overall financial situation of the enterprise. There are three main ways for tax reduction to improve enterprise performance: First, the reduction of income tax can reduce the cost of capital of enterprises, thus encouraging enterprises to increase production investment and research and development, thus promoting productivity improvement; Second, after the reduction of income tax, the take-home profits of enterprises will increase, and more cash will be available for production investment and research and development (24). Third, the reduction of income tax means that the after-tax income of unit input is rising, which will produce good social benefits and benefit the development of enterprises. The effect mechanism of turnover tax reduction and income tax reduction on enterprise performance is basically the same, but income tax enterprises generally cannot be transferred, while turnover tax can be transferred (25, 26).

From the perspective of influencing factors, there are four main aspects that will affect the financial performance of enterprises: First, the disclosure of corporate social responsibility, Subin and Yuan (27) and Wenjie (28) find that corporate social responsibility disclosure and financial performance have a promoting relationship; Second, the political connection of enterprises, Chuan et al. (29) shows that corporate political connections can promote financial performance; Third, technological innovation, Xuan et al. (30) find that in the pharmaceutical industry, technological innovation has a significant role in promoting financial performance. Naiping et al. (31) believe that technological innovation would have a positive impact on the short-term and long-term financial performance of enterprises; Fourth, the corporate governance structure, Chenggang et al. (32) studies the data of listed companies through empirical methods, the result shows that the quality of corporate treatment structure has a promoting effect on financial performance, and there is no significant difference between state-owned enterprises and non-state-owned enterprises.

Considering the time value of enterprise development, this paper attempts to analyze the different situations of financial performance in the short term and long term. In the short term, for the enterprises enjoying the tax reduction policy, their cash flow will increase immediately, which will play a significant buffer role for those enterprises with financing problems. Enterprises can make use of the increased cash flow input through tax reduction policies in all aspects of enterprise production and operation, such as increasing investment and improving the weakness of enterprise shortage. These measures will promote the performance of the financial performance of enterprises. In the long run, we can make corresponding conjectures according to the theory of signal transmission. As the government has adopted tax reduction policies for enterprises, it will make investors more confident to invest so as to alleviate the financing problems faced by enterprises to a certain extent. In particular, the continuation of the government's tax reduction policy will probably keep this kind of investment experience, so as to provide cash flow for the sustainable development of enterprises, and thus improve the long-term financial performance of enterprises. In addition, because the design principles of different taxes are different, we speculate that the specific impact of different taxes on financial performance is also different. Income tax is levied on the consequences of the end of the transaction because the enterprise is the ultimate undertaker, so they cannot pass on this tax, directly reduce the cash flow of the enterprise, and reduce the profitability of the enterprise. Commodity turnover tax is a kind of tax levied on the behavior of commodity market transactions. Although the tax will also reduce the cash flow of enterprises, the transaction does not end. Enterprises can transfer the commodity turnover tax as a part of production cost to consumers in a certain proportion, so the impact of commodity turnover tax on enterprises is weakened. Therefore, the commerce turnover tax levies the transaction behavior, while the income tax levies the transaction result income, which has a different influence on the financial performance of the enterprise. Therefore, the preferential benefits obtained in the income tax can all affect the enterprise itself, and because of the transferability of the commodity turnover tax, the preferential benefits brought by the commodity turnover tax will be enjoyed by both the enterprise and the consumer, but the proportion of the two parties is different, and the size of this proportion is mainly determined by the elasticity of commodity demand. We make the following hypothesis:

Hypothesis 1: Income tax reduction is more conducive to the improvement of short-term financial performance;
Hypothesis 2: Compared with short-term financial performance, turnover tax reduction policy is more beneficial to improve long-term financial performance of enterprises.

For enterprises with different types of ownership, there may also be inconsistencies in the implementation of tax reduction policies. First, due to the influence of traditional Chinese concepts. For state-owned enterprises, because of their status as state-owned enterprises, more and more high-end talents will choose to work in state-owned enterprises, which leads to the overall quality of financial staff in state-owned enterprises is better than that in non-state-owned enterprises; Second, compared with other enterprises, state-owned enterprises will be more strict and standardized in government guidance, so financial management business is more scientific and effective than non-state-owned enterprises; Third, because the financial strength of state-owned enterprises is relatively stronger in general, they will be more likely to actively use modern science and technology to strengthen financial supervision and optimize financial workflow.

China has a vast territory, and there are distinct regional differences in economic development. Due to policy factors, factor endowments, regional factors, infrastructure and other factors, China finally shows regional differences in economic development. Compared with the central and western regions, the eastern region has a better business environment for enterprises, with advantages in geographical location, human resources, economic strength, openness and national policies. Furthermore, resource-based theory represented by Penrose believes that an enterprise can be regarded as a collection of various resources under the management framework, and the efficiency of an enterprise is jointly determined by the investment in resources and the management of resources. Due to a long history of inherent deficiency in economic development level and other aspects in the Midwestern China, these may lead to mismatch between the capability of management in companies' resources and the strength of investment in resources, and it is difficult to coordinate resources of information and human. Therefore, the Midwestern China is more responsive and effective to the tax statutory deduction.

For the above reasons, we propose the following hypothesis:

Hypothesis 3: Tax reduction policy has a better effect on the financial performance of non-state-owned enterprises than state-owned enterprises;
Hypothesis 4: Tax reduction policy plays a more significant role in promoting the financial performance of enterprises in central and western Regions.

## Empirical Analysis

### Data Sources

Pharmaceutical manufacturing industry is a key industry related to national livelihood. “Made in China 2025” takes the development of pharmaceutical industry as a key content. As an important force of public health security, it is of great practical significance to analyze the impact of tax reduction policies on their financial performance. In this paper, we select the data of 241 A-share listed pharmaceutical manufacturing enterprise from 2008 to 2020 after filtering some samples. The data are obtained from CSMAR and WIND. The main reasons for choosing the data of listed companies as the research object are as follows: First, the development of listed companies is relatively mature, and the financial system is relatively complete; second, due to its open and transparent data in all aspects, data indicators are relatively comprehensive and reliable, and data acquisition is relatively easy. In order to avoid the impact of information disclosure on the results of empirical research, this study selects data according to the following original: (1) Remove all ST Companies in 2008–2020; (2) Remove companies with incomplete data and incomplete data; (3) Remove samples with annual income tax and commodity turnover tax <0; (4) Use the method of tail reduction to remove 1% of the value at both ends of each index.

### Variables Setting

#### Dependent Variable

As shown in Table 2, in the aspect of the financial performance of enterprises, in order to better reflect the effect of tax reduction on the financial performance of enterprises in time, financial performance is divided into short-term and long-term financial performance in this study. According to the experience of most papers, ROE is used to express short-term financial performance, and Tobin's Q value is selected to express long-term financial performance.

**Table 2:** Variables setting.

**Type of variable**	**Variable code**	**Variable name**	**Calculation method**
Dependent variables	TBQ	Tobin's Q value	Market value / replacement cost of the company
	ROE	Return on net assets	Net profit / average net assets
	ROA	Return on total assets	Net profit / total assets
Independent variables	etr	Actual tax bearing rate of income tax	Income tax / EBIT actually paid
	ctr	Actual tax bearing rate of turnover tax	(Actual taxes paid—Actual income tax paid) / total assets
Control variables	zczj	Total assets	Total assets at the end of the year
	birth	Enterprise age	2022—year of establishment
	zcmjd	Asset intensity	Net fixed assets / total assets
	chmjd	Inventory density	Net amount of inventory / total assets
	lev	Leverage ratio	Total indebtedness / total assets

The reason why the return on net assets is chosen to express the short-term financial performance is that the most important position of enterprise operation is profit, and the maximization of profit is the best embodiment of the effectiveness of the company's financial management. Therefore, we can use the return on net assets to reflect the above purpose. In addition, in many studies, the return on equity is used as the most comprehensive indicator, which can comprehensively measure the financial performance of different enterprises.

The reason why Tobin's Q value is selected is to use it to express long-term financial performance because this indicator is generally obtained by the ratio of the market value of the company to its own replacement cost, which can well measure the future development trend of the enterprise to a certain extent. Compared with the short-term financial indicators, Tobin's Q value is not easy to be manipulated by the enterprise itself. It can not only predict the future cash flow of the enterprise but also integrate the market value and profitability to reflect the financial performance of the enterprise.

#### Independent Variables

Based on the experience of most articles, in this paper, the actual tax bearing rate (etr) of corporate income tax and the actual tax bearing rate (ctr) of commodity turnover tax are taken as independent variables. It can be found that the lower the tax bearing rate is, the greater the tax reduction effort and the greater the preferential range of tax.

(1) The actual tax bearing rate (etr) of income tax reflects the real degree of the income tax preference enjoyed by the enterprise itself. The lower the actual tax bearing, the deeper the discount. In the research of this paper, the actual tax bearing rate of income tax is obtained by comparing the total amount of income tax actually paid by the enterprise with the total amount of profit before interest and tax of that year.

(2) For the actual tax bearing rate (ctr) of the commodity turnover tax, the enterprise business tax, and surcharges are used to represent the total amount of the commodity turnover tax, and then the ratio of the total business income is obtained. In this paper, the second method is used to calculate the turnover tax (ctr).

**Figure 2:**
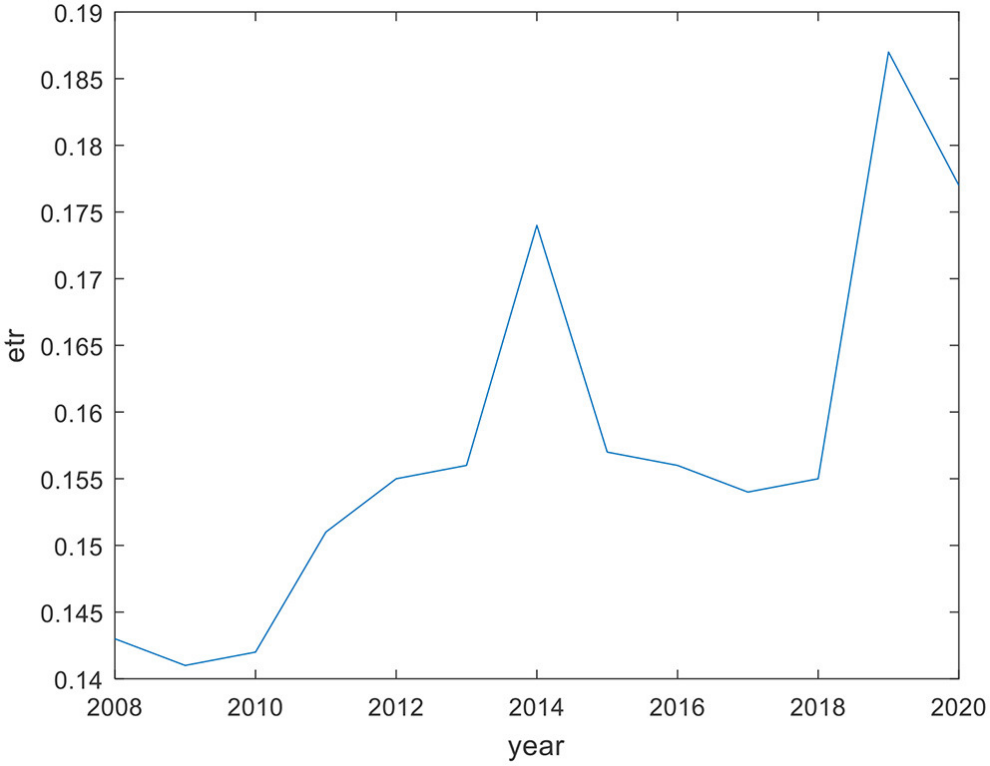
Actual tax bearing rate of income tax (2008–2020).

**Figure 3:**
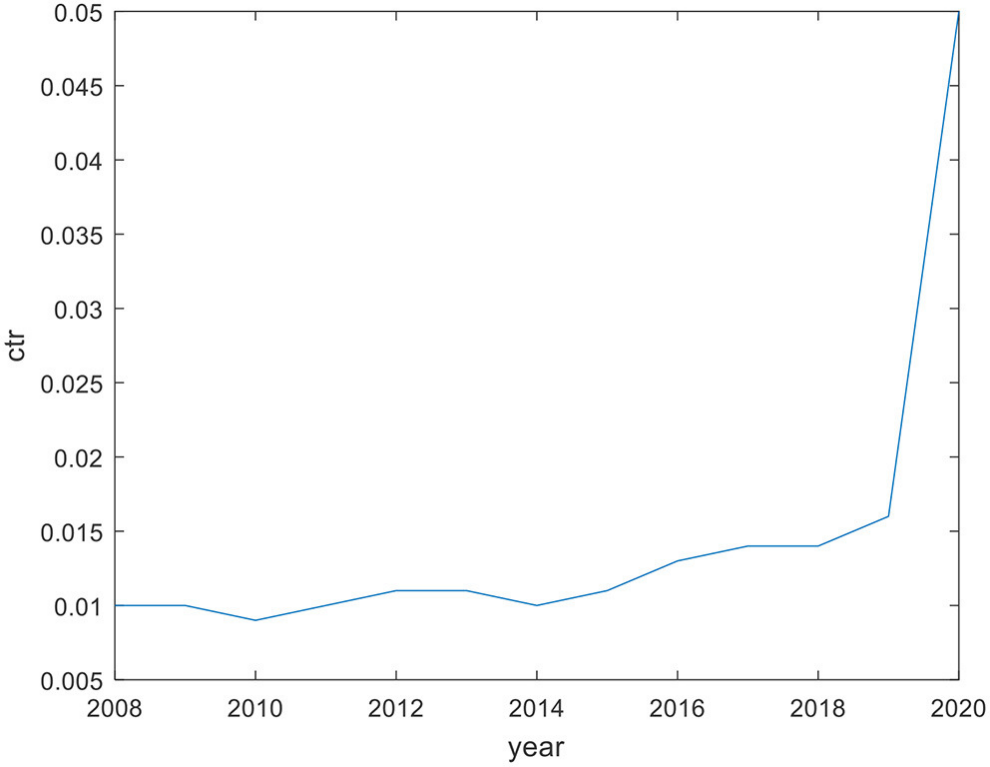
Actual tax bearing rate of commodity turnover tax (2008–2020).

It can be seen from Figures 2, 3 that in the first 5 years, on the whole, the actual tax bearing rate of income tax has increased to a certain extent, but it is far lower than the statutory income tax rate of 25%. The actual tax bearing rate of turnover tax is stable on the whole, and is below 2% in all years except 2020, which may be affected by COVID-19.

#### Control Variables

Referring to the literature published by Chang and Yiling (2), Weibao (33), Qing (34) and other scholars, the following control variables are selected in this paper: Enterprise size (zczj), the size of an enterprise may produce scale effects; Leverage ratio (lev), the higher the asset-liability ratio, the greater the financial leverage effect; Asset intensity (zcmjd), the higher the intensity of the asset, the relative profits will be averaged out and therefore the smaller the profits will be; Inventory intensity (chmjd), inventory intensity directly affects the management cost of enterprises; Enterprise age (birth), the longer the enterprise is established, the more abundant the manpower, material resources and financial resources.

### Model of Measurement

Considering that there will be some impacts that do not change with time on the dependent variables, we establish the following two individual fixed effect regression models to analyze the possible impact of tax reduction policies on the financial performance of enterprises:


(1) $$\begin{array}{r} ROE_{it=}\beta_{0}+\beta_{1}etr_{it}+\beta_{2}ctr_{it}+\beta_{3}X_{it}+\tau_{i}+u_{it} \end{array}$$

(2) $$\begin{array}{r} TBQ_{it=}\beta_{0}+\beta_{1}etr_{it}+\beta_{2}ctr_{it}+\beta_{3}X_{it}+\tau_{i}+u_{it} \end{array}$$

In the first model, we test the possible impact of the actual tax bearing rate of income tax and the actual tax bearing rate of commodity turnover tax on the short-term financial performance return on net assets of enterprises. In the second model, it is used to test the possible impact of different tax cuts on the long-term financial performance of enterprises.

### Regression Analysis

#### Descriptive Analysis

Table 3 describes the mean, standard error, minimum and maximum values of each indicator in the sample. In order to avoid possible heteroscedasticity, the total value of assets and the age of the enterprise are logarithmic. It can be seen from the table that the average ROE is 0.14, the overall level is not high, and the difference between the maximum value and the minimum value is small, indicating that the difference between the ROE of the sample enterprises is small. The average value of TBQ is 2.63, indicating that the market value of different enterprises varies greatly. The value range of etr and ctr varies greatly, indicating that the difference of actual tax bearing rate of income tax and actual tax bearing rate of turnover tax is prominent among different enterprises.

**Table 3:** Description of variables.

	**(1)**	**(2)**	**(3)**	**(4)**	**(5)**
**Variables**	**N**	**Mean**	**SD**	**Min**	**Max**
ROE	2,325	0.146	0.129	0.000	1.986
TBQ	2,092	2.633	1.820	0.761	22.572
etr	1,999	0.160	0.184	0.000	6.824
ctr	2,157	0.0166	0.164	0.000	6.986
lev	2,160	0.321	0.202	0.008	1.893
zcmjd	2,159	0.217	0.117	0.007	0.863
chmjd	2,160	0.118	0.0981	0.003	0.719
lnzczj	2,160	21.74	0.972	19.21	25.15
lnbirth	3,316	2.667	0.486	0.693	3.689

#### Correlation Analysis

Table 4 shows the degree of correlation between each variable. It can be seen from the table that the absolute values of correlation coefficients between the two independent variables and the four control variables are far <0.4, and the VIF values calculated are all <2. Therefore, the multicollinearity problem between the independent variables and the control variables can be excluded. In addition, ROE has a significant negative linear correlation with etr, lnbirth, lev, zcmjd and chmjd. TBQ had significant negative linear correlation with lnzczj, lev, zcmjd, and positive linear correlation with lnbirth?

**Table 4:** Correlation of variables.

	**ROE**	**TBQ**	**ETR**	**CTR**	**lnzczj**	**lnbirth**	**lev**	**zcmjd**	**chmjd**
ROE	1								
TBQ	0.319^***^	1							
etr	−0.067^***^	−0.00700	1						
ctr	−0.0260	−0.0100	0.088^***^	1					
lnzczj	0.0280	−0.168^***^	0.0340	−0.0130	1				
lnbirth	−0.222^***^	0.082^***^	0.084^***^	−0.00400	0.336^***^	1			
lev	−0.065^***^	−0.138^***^	−0.0130	0.0200	0.183^***^	0.129^***^	1		
zcmjd	−0.140^***^	−0.070^***^	−0.0300	−0.0190	−0.134^***^	−0.0150	0.262^***^	1	
chmjd	−0.039^*^	−0.00400	0.00200	−0.0210	0.052^**^	0.056^***^	0.263^***^	−0.051^**^	1

*t-statistics in parentheses*.

*** *p < 0.01*,

** *p < 0.05*,

* *p < 0.1*.

#### Regression Analysis

Hausman test of fixed effects and random effects was conducted on models (1) and (2), and the test results rejected the null hypothesis, indicating that the fixed effects model should be adopted. As can be seen from Table 5, the actual tax bearing rate of income tax is negatively and significantly correlated with short-term financial performance, and positively but not significantly correlated with the actual tax bearing rate of turnover tax, indicating that income tax reduction can significantly promote the improvement of short-term financial performance of enterprises, while the relationship between turnover tax and short-term financial performance of enterprises is not obvious. The actual tax bearing rate of turnover tax is significantly negatively correlated with the long-term financial performance of enterprises, while the actual tax bearing rate of income tax is not significantly correlated with the long-term financial performance of enterprises. After the addition of control variables, the above conclusion is still valid, so the hypothesis 1 and the hypothesis 2 are verified. In addition, enterprise size has a significant positive effect on short-term performance, but is not conducive to the improvement of long-term financial performance. The longer the enterprise is established, it has a significant promoting effect on long-term financial performance. Leverage ratio has a significant effect on short-term financial performance, but not on long-term financial performance.

**Table 5:** Principal regression result.

	**(1)**	**(2)**	**(3)**	**(4)**
**Variables**	**ROE**	**ROE**	**TBQ**	**TBQ**
etr	−0.033^***^	−0.032^***^	−0.179	−0.172
	(−2.73)	(−2.59)	(−1.07)	(−1.04)
ctr	0.290	0.287	−13.953^**^	−18.205^***^
	(0.84)	(0.80)	(−2.57)	(−3.30)
lnzczj		0.017^***^		−0.487^***^
		(2.69)		(−4.94)
lnbirth		−0.015		2.140^***^
		(−0.54)		(4.90)
lev		0.046^**^		0.114
		(2.14)		(0.38)
zcmjd		−0.041		−0.115
		(−1.39)		(−0.25)
chmjd		−0.028		1.145^**^
		(−0.71)		(1.96)
Constant	0.160^***^	−0.157	1.960^***^	6.856^***^
	(15.33)	(−1.10)	(12.55)	(3.06)
id FE	YES	YES	YES	YES
year FE	YES	YES	YES	YES
Observations	1,971	1,956	1,933	1,921
R–squared	0.035	0.046	0.130	0.158
Number of id	254	250	248	244

*t-statistics in parentheses*.

*** *p < 0.01*,

** *p < 0.05*.

#### Heterogeneity Analysis

As can be seen from Table 6, income tax reduction has a significant promotion effect on short-term financial performance of non-state-owned enterprises, but not for state-owned enterprises. It has a significant promotion effect on enterprises in the central and western regions, but not for the eastern regions. Turnover tax reduction has no significant promoting effect on non-state-owned enterprises, state-owned enterprises, central and western enterprises and eastern enterprises, and may even have significant negative effect.

**Table 6:** Results of heterogeneous regression of short–term financial performance.

	**(1)**	**(2)**	**(3)**	**(4)**
**Variables**	**ROE-s0**	**ROE-s1**	**ROE-a0**	**ROE-a1**
etr	−0.220^***^	−0.016	−0.325^***^	−0.017
	(−4.65)	(−1.48)	(−5.20)	(−1.53)
ctr	0.162	2.337^**^	0.605	0.040
	(0.41)	(2.23)	(0.67)	(0.11)
lnzczj	0.021^***^	0.026^**^	0.032^**^	0.002
	(2.81)	(2.02)	(2.56)	(0.36)
lnbirth	0.013	−0.062	−0.098	0.022
	(0.41)	(−1.01)	(−1.51)	(0.78)
lev	0.054^**^	−0.027	0.086^**^	0.041^*^
	(2.07)	(−0.74)	(2.15)	(1.67)
zcmjd	−0.045	−0.088^*^	0.057	−0.165^***^
	(−1.27)	(−1.68)	(1.15)	(−4.58)
chmjd	−0.036	0.053	−0.197^***^	0.083^*^
	(−0.82)	(0.63)	(−2.95)	(1.84)
Constant	−0.246	−0.283	−0.233	0.074
	(−1.43)	(−1.05)	(−0.80)	(0.47)
id FE	YES	YES	YES	YES
year FE	YES	YES	YES	YES
Observations	1,507	449	755	1,201
R-squared	0.074	0.067	0.091	0.086
Number of id	209	41	92	158

*t-statistics in parentheses*.

*** *p < 0.01*,

** *p < 0.05*,

* *p < 0.1*.

As can be seen from Table 7, income tax reduction has no significant effect on the long-term financial performance of enterprises in non-state-owned or state-owned, central and western regions or eastern regions. Turnover tax reduction plays a significant role in promoting the long-term financial performance of both non-state-owned and state-owned enterprises, especially for state-owned enterprises. It also plays a significant role in promoting the long-term financial performance of enterprises in central and western regions, but not in the east.

**Table 7:** Results of heterogeneous regression of long–term financial performance.

	**(1)**	**(2)**	**(3)**	**(4)**
**Variables**	**TBQ-s0**	**TBQ-s1**	**TBQ-a0**	**TBQ-a1**
etr	0.212	−0.224	−0.602	−0.184
	(0.60)	(−1.33)	(−0.77)	(−1.04)
ctr	−12.428^**^	−65.485^***^	−52.193^***^	−8.060
	(−2.03)	(−3.95)	(−4.78)	(−1.21)
lnzczj	−0.507^***^	−0.634^***^	−0.556^***^	−0.467^***^
	(−4.37)	(−3.07)	(−3.74)	(−3.51)
lnbirth	1.843^***^	2.178^*^	3.554^***^	1.878^***^
	(3.72)	(1.94)	(4.47)	(3.49)
lev	0.309	−0.736	−0.010	0.297
	(0.87)	(−1.26)	(−0.02)	(0.66)
zcmjd	0.215	−0.654	−0.886	0.460
	(0.40)	(−0.79)	(−1.45)	(0.69)
chmjd	0.901	0.677	1.047	0.886
	(1.35)	(0.51)	(1.32)	(1.06)
Constant	7.818^***^	10.605^**^	5.054	7.021^**^
	(2.93)	(2.33)	(1.41)	(2.35)
id FE	YES	YES	YES	YES
year FE	YES	YES	YES	YES
Observations	1,470	451	748	1,173
R–squared	0.150	0.249	0.230	0.143
Number of id	203	41	91	153

*t-statistics in parentheses*.

*** *p < 0.01*,

** *p < 0.05*,

* *p < 0.1*.

Based on the above analysis, the hypothesis 3 is partially verified and the hypothesis 4 is fully verified. The possible reason why turnover tax reduction plays a greater role in promoting the long-term performance of state-owned enterprises than non-state-owned enterprises is that state-owned enterprises have abundant capital and shoulder important tasks of national economy and people's livelihood. They are not eager to improve short-term financial performance and pay more attention to long-term effects in enterprise development strategy.

#### Test of Robustness

In order to verify the stability of the results, this paper carries out tests from the following two aspects.

First, the dependent variable was replaced, and return on assets (ROA) was used to replace return on equity (ROE) for regression. Regardless of whether control variables are added, the regression results are consistent and significant with the original regression results.

Second, shorten the data period. Considering that the research object of this paper is the medical manufacturing industry, the tax and fee reduction policies for the manufacturing industry are relatively stable from 2016 to 2020 without major policy changes, so the original data period is shortened to 2016–2020. Regardless of whether control variables are added, the regression results are consistent and significant with the original regression results.

#### Further Analysis

In the previous analysis, we believed that there was a linear relationship between tax reduction policy and enterprise performance, without considering the possibility of non-linear relationship. Then, we added the quadratic term of tax reduction policy index to conduct regression, and the results are shown in Table 8.

**Table 8:** Regression results of adding quadratic terms.

	**(1)**	**(2)**	**(3)**	**(4)**
**Variables**	**ROE**	**TBQ**	**ROE**	**TBQ**
etr	−0.215^***^	−0.341		
	(−5.65)	(−0.83)		
etr^*^etr	0.029^***^	0.025		
	(5.10)	(0.37)		
ctr			1.354^**^	−27.841^***^
			(2.13)	(−3.05)
ctr^*^ctr			−8.483^*^	83.192
			(−1.89)	(1.23)
Constant	−0.128	7.409^***^	−0.103	8.890^***^
	(−0.90)	(3.31)	(−0.73)	(3.39)
Control variables	YES	YES	YES	YES
id FE	YES	YES	YES	YES
year FE	YES	YES	YES	YES
Observations	1,958	1,923	1,988	2,076
R-squared	0.060	0.153	0.041	0.173
Number of id	250	244	250	252

*t-statistics in parentheses*.

*** *p < 0.01*,

** *p < 0.05*,

* *p < 0.1*.

According to the above regression results, after using the U-shaped test, short-term corporate performance is positively U-shaped with the actual tax bearing rate of income tax, while the statistical results show that the number of samples with the actual tax bearing rate of income tax greater than the extreme point is very small (1/1958), which can be ignored. Therefore, it can be considered that there is no non-linear relationship. In addition, by using the same method, we find that the actual tax bearing rate of turnover tax is in an inverted U-shaped with short-term corporate performance. After statistics, we find that the number of samples with the actual tax bearing rate of turnover tax greater than the extreme point is very small (11/1,988), which can be ignored. Therefore, it can be considered that there is no non-linear relationship. Based on the above analysis, we believe that the nonlinear relationship is not tenable.

In addition, OLS is the most commonly used regression method in empirical studies. Standard least squares linear regression only focuses on the influence of E(Y|X). In empirical, however, many researchers on the distribution of Y | X other quantile is also very interested in, so we used quantile regression method, under the actual tax rate at different quantiles of regression, the results as shown in Table 9.

**Table 9:** Quantile regression results.

**Dependent varables**	**Independent variables**	**OLS**	**Quantile regression**				
			**0.1**	**0.3**	**0.5**	**0.7**	**0.9**
ROE	etr	−0.031^**^	−0.177^*^	0.051	0.229	0.011	−0.008
		(−2.57)	(−1.92)	(−0.05)	(−1.12)	(−0.03)	(−0.01)
	ctr	0.365	−0.184	2.674	1.185	1.184	0.363
		(1.06)	(−0.15)	(−0.41)	(−1.61)	(−0.11)	(−0.15)
TBQ	etr	−0.245	−0.59	−0.043	−0.181	−0.082	0.187
		(−1.39)	(−0.65)	(−0.07)	(−0.08)	(−0.02)	(−0.03)
	ctr	−23.676^***^	0.87	0.254	0.367	3.843	−0.291
		(−4.32)	(−0.14)	(−0.01)	(−0.06)	(−0.07)	(−0.01)

*t-statistics in parentheses*.

*** *p < 0.01*,

** *p < 0.05*,

* *p < 0.1*.

As shown in Table 8, in OLS regression, income tax reduction can significantly improve short-term corporate performance, while in quantile regression, only when the income tax bearing rate is in the low quantile can it significantly improve short-term financial performance. In OLS regression, turnover tax reduction has a significant improvement effect on long-term corporate performance, while in quantile regression, each sub point of actual turnover tax bearing rate has no significant effect on long-term financial performance. Therefore, we believe that among tax reduction policies, income tax reduction is better.

## Conclusions And Recommendations

### Conclusions

Based on the research of listed pharmaceutical manufacturing enterprises in China, this paper analyzes the specific impact of tax reduction of different tax types and heterogeneous enterprises on tax reduction policies, and draws the following conclusions:

First, the implementation of tax reduction policies can really play a positive role in the financial performance of enterprises. Due to the differences in the collection principles of different taxes, the impact of varying tax reduction policies on the short-term and long-term financial performance of enterprises will be inconsistent. The reduction of income tax significantly improves the short-term financial performance of enterprises, while the reduction of commodity turnover tax significantly enhances the long-term financial performance of enterprises.

Second, the implementation of tax reduction policy will significantly promote the financial performance of both non-state-owned and state-owned enterprises. Due to the different social status of enterprises with different ownership, tax reduction policy has a better effect on the financial performance of non-state-owned enterprises than state-owned enterprises.

Third, on the whole, enterprise size is beneficial to short-term financial performance, not to long-term financial performance. For enterprises in central and western regions, the promotion effect is significant, but not for enterprises in eastern regions.

Fourth, the establishment time of an enterprise has no significant effect on short-term financial performance, but significantly promotes long-term financial performance.

Fifth, corporate leverage plays a significant role in promoting short-term financial performance, but not in promoting long-term financial performance. Leverage ratio plays a significant role in promoting the short-term financial performance of non-state-owned enterprises, but not for state-owned enterprises.

### Recommendations

Based on the above conclusions, we put forward some suggestions as follows:

First, the state should maintain the continuity of tax reduction policy to benefit its positive role better and enable it to play its role continuously.

Second, for some enterprises facing short-term difficulties, the state can make more efforts in the aspect of income tax reduction to help them get through the problems.

Third, for enterprises in the process of sound development, when considering their sustainable development, the state should further explore and deepen the tax reduction policy of turnover tax, so that enterprises can focus on the formulation of long-term development strategy.

Fourth, we need to pay attention to the balance of tax reduction policies between non-state-owned enterprises and state-owned enterprises. Because state-owned enterprises have many advantages such as congenital position, resources, and power, non-state-owned enterprises have their own defects in the process of development. Therefore, the state needs to refine further “structural tax reduction” measures, and combine “inclusive tax reduction” with them, so as to play a better role.

Fifth, the size of the enterprise is not the bigger the better, when the enterprise is large, there may be various risks. The development of enterprises should not only focus on the scale of enterprises, but also control the size of enterprise development, and use more resources to improve the “high quality” development of enterprises.

Sixth, enterprises can maintain a moderate leverage ratio, but to achieve long-term and stable development of enterprises, they should not rely on the leverage ratio. Instead, they should try their best to improve and boost the internal strength and achieve innovation-driven development.

## Data Availability Statement

The original contributions presented in the study are included in the article/supplementary material, further inquiries can be directed to the corresponding author.

## Author Contributions

All authors listed have made a substantial, direct, and intellectual contribution to the work and approved it for publication.

## Funding

This work was sponsored in part by the Center of Scientific and Technological Innovation and New Economy Institute of Chengdu-Chongqing Economic Zone (No: CYCX202011).

## Conflict of Interest

The authors declare that the research was conducted in the absence of any commercial or financial relationships that could be construed as a potential conflict of interest.

## Publisher's Note

All claims expressed in this article are solely those of the authors and do not necessarily represent those of their affiliated organizations, or those of the publisher, the editors and the reviewers. Any product that may be evaluated in this article, or claim that may be made by its manufacturer, is not guaranteed or endorsed by the publisher.
